# A Bifunctional Synthetic Peptide With Antimicrobial and Plant Elicitation Properties That Protect Tomato Plants From Bacterial and Fungal Infections

**DOI:** 10.3389/fpls.2021.756357

**Published:** 2021-10-18

**Authors:** Laura Montesinos, Beatriz Gascón, Lidia Ruz, Esther Badosa, Marta Planas, Lidia Feliu, Emilio Montesinos

**Affiliations:** ^1^Institute of Food and Agricultural Technology-CIDSAV-XaRTA, University of Girona, Girona, Spain; ^2^LIPPSO, Department of Chemistry, University of Girona, Girona, Spain

**Keywords:** bifunctional peptide, antimicrobial, plant defense elicitor, plant disease, tomato

## Abstract

The hybrid peptide BP178 (KKLFKKILKYLAGPAGIGKFLHSAKKDEL-OH), derived from BP100 (KKLFKKILKYL) and magainin (1–10), and engineered for plant expression, had a strong bactericidal activity but not fungicidal. Moreover, the preventive spray of tomato plants with BP178 controlled infections by the plant pathogenic bacteria *Pseudomonas syringae* pv. tomato and *Xanthomonas campestris* pv. vesicatoria, as well as the fungus *Botrytis cinere*a. The treatment of tomato plants with BP178 induced the expression of several genes according to microarray and RT-qPCR analysis. Upregulated genes coded for several pathogenesis-related proteins, including PR1, PR2, PR3, PR4, PR5, PR6, PR7, PR9, PR10, and PR14, as well as transcription factors like ethylene transcription factors, WRKY, NAC and MYB, involved in the salicylic acid, jasmonic acid, and ethylene-signaling pathways. BP178 induced a similar gene expression pattern to flg15 according to RT-qPCR analysis, whereas the parent peptide BP100 did not trigger such as a strong plant defense response. It was concluded that BP178 was a bifunctional peptide protecting the plant against pathogen infection through a dual mechanism of action consisting of antimicrobial activity against bacterial pathogens and plant defense elicitation on plant host.

## Introduction

Chemical control with conventional pesticides is an important part of the management of bacterial and fungal diseases of plant crops, but their extensive use has a negative environmental impact and often results in the emergence of resistance within the pathogen population (McManus et al., 2002; Brent and Hollomon, 2007; Sundin et al., 2016). Biological control appears to be an alternative or complement to the use of chemical pesticides, and several bacterial and fungal strains are commercialized as microbial biopesticides (Johnson and Temple, 2013; Montesinos and Bonaterra, 2017). Similarly, nonmicrobial biopesticides offer great possibilities for a sustainable disease management, and antimicrobial peptides (AMPs) have been proposed as novel pesticides to overcome problems due to fungal and bacterial plant pathogens (Montesinos et al., 2012; Zeitler et al., 2013; Datta et al., 2015; Badosa et al., 2017; Li et al., 2021). In addition, the conventional management of plant bacterial and fungal diseases has been based on targeting directly plant pathogens, but considerable efforts are oriented to identify compounds that activate the immune system of the plant (Tripathi and Dubey, 2004; Reignault and Walters, 2007; Thakur and Sohal, 2013; Abdul Malik et al., 2020). Thus, crop disease protection is currently oriented to a multitarget approach, consisting of pathogen inactivation and plant defense stimulation.

Plants have evolved several defense strategies to protect themselves from biotic and abiotic stresses (Montesano et al., 2003; Nejat and Mantri, 2017; Lamers et al., 2020). These responses include a set of induced mechanisms at the tissular level, like the rapid and localized cell death, termed hypersensitive response, and the production and accumulation of near 17 families of pathogenesis-related (PR) proteins (van Loon et al., 1994; Christensen et al., 2002; Jiang et al., 2015). PR expression is known to be regulated by defense or stress-signaling molecules and by abiotic agents (Jiang et al., 2015). In addition, plants have the ability to recognize microbe-associated molecular patterns (MAMPs) or pathogen-associated molecular patterns (PAMPs) that trigger a cascade of reactions conferring disease resistance (Albert, 2013; Beunouaret et al., 2014). Some examples of MAMPs/PAMPs include bacterial flagellin, peptidoglycans, lipopolysaccharides, cell wall glucans, fungal chitin, and sterols, among several compounds (Mishra et al., 2012; Gao et al., 2015). These MAMPs are recognized by pattern recognition receptors (PRRs) and elicit basal resistance referred to as PAMP/MAMP-triggered immunity (PTI-MTI) (Ausubel, 2005; Newman et al., 2013; Gao et al., 2015; Saijo et al., 2018). Apart from microbial elicitors, plants sense damage-associated molecular patterns (DAMPs), a plant-derived type of molecules like systemin (Boller and Felix, 2009; Albert, 2013). Besides the induction of locally restricted responses, plants have the ability to induce systemic defense responses, the so-called systemic acquired resistance (SAR) and induced systemic resistance (ISR), generally termed as induced resistance (IR). IR involves three main signaling transduction pathways, mediated by the phytohormones salicylic acid (SA), jasmonic acid (JA), and ethylene (Park et al., 2007; Rivas-San and Plasencia, 2011; Pieterse et al., 2014; Dhar et al., 2020). The application of chemical or biological elicitors to plants (e.g., harpins, acibenzolar-*S*-methyl, and fosetyl-Al) has been reported to protect plants from biotic stresses (Bektas and Eulgem, 2015; Badosa et al., 2017).

In the past years, there has been intensive research to identify plant defense elicitors from natural origin, and several functional peptides have been reported. This is the case of bacterial flagellin, which has been shown to act as a plant defense elicitor (Meindl et al., 2000), because the perception of bacterial flagellin by plant cells leads to the induction of defense-related genes followed by an oxidative burst, callose deposition, and ethylene production (Gómez-Gómez and Boller, 2002). Interestingly, analogs of flagellin (flg22 or flg15) and several natural or synthetic peptides were reported to trigger innate immunity in plants (Meindl et al., 2000; Brotman et al., 2009; Wei et al., 2017; Czékus et al., 2021). In this context, flg15 induced ROS production and the expression of several genes involved in salicylic acid, jasmonic acid, and ethylene-signaling pathways in tomato plants (Robatzek et al., 2007; Caravaca-Fuentes et al., 2021).

Our group has developed several families of peptides derived from natural compounds or *de novo* designed. Our goal was to find short sequences with high antimicrobial activity, low toxicity, and high stability to protease degradation (Montesinos et al., 2012). In particular, we designed and synthesized a library of linear undecapeptides (CECMEL11) (Ferré et al., 2006; Badosa et al., 2007), from which we identified sequences with an excellent biological activity profile that have been used successfully to control diseases caused by fungal and bacterial plant pathogens of economic importance (Badosa et al., 2007, 2009; Baró et al., 2020). Several peptide conjugates from members of the CECMEL11 library, like BP358 (containing flg15 and BP16), showed antimicrobial and plant defense elicitation activities in the *Erwinia amylovora*/pear pathosystem (Caravaca-Fuentes et al., 2021).

In addition, we designed a family of hybrid peptides to be produced in plant systems. Among them, BP178 (KKLFKKILKYL-AGPA-GIGKFLHSAK-KDEL-OH), incorporating BP100 (KKLFKKILKYL), magainin (1–10), an AGPA hinge for connecting both, and a KDEL endoplasmic reticulum retention signal, exhibited a strong bactericidal effect against several plant pathogenic bacteria and a very slight toxicity, but gave an HR-type reaction in tobacco leaves (Badosa et al., 2013). The peptide was expressed in the transgenic rice seed endosperm and protected seedlings from bacterial infection, but the protective effect was not completely explained by its antimicrobial properties (Montesinos et al., 2017).

In the present study, we planned to elucidate the mechanism of action of BP178 and whether it is able to trigger plant defense responses in tomato as a model plant. Specifically, the aim of this work was to determine if the topical application of the peptide to plants (1) protects against bacterial and fungal infection and (2) induces defense and stress-related gene expression. The effect of BP178 was compared to the plant defense elicitor peptide flg15, which has no antimicrobial activity, and to the parent bactericidal undecapeptide BP100 with bactericidal but no defense elicitor activity.

## Materials and Methods

### Bacterial and Fungal Strains and Growth Conditions

The bacterial pathogens *Xanthomonas campestris* pv. vesicatoria Xcv206 (Xcv) (D. F. Ritchie, Department of Plant Pathology, North Carolina State University) and *Pseudomonas syringae* pv. tomato DC3000 (Pto) (J. Murillo, Plant Pathology, Public University of Navarra, Spain), and the necrotrophic fungus *Botrytis cinerea* (Bc) (CECT 20518) were used. Bacterial strains were cultured in LB agar for 24 h at 28°C and scrapped from the surface to prepare suspensions adjusted to 10^8^ CFU/ml. Bc was grown on potato dextrose agar (PDA) for 10 days at 23°C. Spores were collected by spreading sterile distilled water containing 0.01% (v/v) tween-20 onto the surface of the plate. The spore suspension was filtered through three layers of sterile cheesecloth and adjusted to 5 × 10^5^ spores/ml.

### Synthesis of Peptides

Peptides BP178 (KKLFKKILKYLAGPAGIGKFLHSAKKDEL-OH), flg15 (RINSAKDDAAGLQIA-OH), and BP100 (KKLFKKILKYL-NH_2_) were synthesized using the solid phase procedure as previously described (Badosa et al., 2007, 2013; Caravaca-Fuentes et al., 2021) (Supplementary Figure 1). An Fmoc-Rink-MBHA resin (0.55 mmol/g) was used for the synthesis of BP100, and a PAC-ChemMatrix resin (0.66 mmol/g) for the synthesis of flg15 and BP178. Once the peptidyl sequences were completed, the resulting resins were treated with trifluoroacetic acid (TFA)/H_2_O/triisopropylsilane (TIS) (95:2.5:2.5) for 2 h at room temperature. Following TFA evaporation and diethyl ether extraction, the crude peptides were dissolved in H_2_O, lyophilized, analyzed by HPLC, and characterized by mass spectrometry. BP178 *t*_R_ = 6.50 min (90% purity); MS (MALDI-TOF) *m/z*: 3,242.7 [M + H]^+^. flg15 *t*_R_ = 5.80 min (>99% purity); MS (ESI) *m*/*z*: 1,542.8 [M + H]^+^. BP100 *t*_R_ = 5.02 min (>99% purity); MS (ESI) *m/z*: 1,421 [M + H]^+^. Lyophilized peptides (acetate salts) were solubilized in double-distilled water to a final concentration of 1 mM and filter sterilized through a 0.2 μm pore Whatman filter. Dilutions of the peptides were made in double-distilled water to obtain the desired final concentrations.

### *In vitro* Antimicrobial Activity of Peptides

Antimicrobial activities were determined using a growth inhibition assay, as described previously (Badosa et al., 2007, 2009). Briefly, 20 μl of each peptide concentration were mixed in a microtiter plate with 20 μl of the suspension of the plant pathogenic bacteria (at final concentration of 10^7^ CFU/ml) and added to 160 μl trypticase soy broth (TBS) (Biòmereux, France). For Bc, 80 μl spore suspension (10^4^ conidia/ml) was mixed with 20 μl of each peptide dilution and 100 μl of double-concentrated PDB to a total volume of 200 μl PDB. Three replicates for peptide and concentration were used. Positive controls containing water instead of peptide and negative controls containing peptide without bacterial/fungal suspension were included. Microplates were incubated at 25°C for 48 h (Pto and Xcv) or 20°C for 6 days (Bc). Microbial growth was determined automatically every hour by optical density measurement at 600 nm (Bioscreen C; Labsystems, Helsinki, Finland) after shaking. The minimal inhibitory concentration (MIC) value was taken as the lowest peptide concentration with no growth at the end of the experiment.

### *In vitro* Bactericidal and Fungicidal Activity of Peptides

Bactericidal activity of the antimicrobial peptides was determined by a contact test or *killing assay*, consisting of the exposure of the target microorganism to an antimicrobial compound for a given time and determining the surviving cells (Lambert, 2004). Twenty μl of the corresponding peptide concentration were mixed in a microtiter plate with 180 μl of bacterial or fungal suspension (at final concentration of 10^7^ CFU/ml for bacteria and 10^4^ CFU/ml for Bc) to a total volume of 200 μl. Three replicates for each concentration, peptide, and pathogen were used. Controls containing water instead of peptide or containing peptide without bacterial/fungal suspension were included. Microplates were incubated at 25°C (Pto and Xcv) or 20°C (Bc) for 1 h. Then, bactericidal activity was assessed through quantification of culturable cells by plate counting and the cell activity was determined using the resazurin method (alamarBlue® cell proliferation and viability reagent, Invitrogen, Thermo Fisher Scientific, Waltham, MA, USA). For bactericidal activity, aliquots of each peptide and concentration were taken and submitted to decimal dilutions, and 20 μl plated onto the surface of LB agar plates. Then, colony forming units (CFU) were quantified at 24–48 h after the incubation at 28°C. Fungicidal activity was determined similarly by spreading 100 μl onto the surface of PDA plates, and CFU were quantified after 7 days of incubation at 23°C. For cell viability measurements, 10 μl of alamarBlue® reagent were mixed with 90 μl of the corresponding microtiter cell suspension at the end of the experiment and transferred to a new microtiter. Incubation was performed for 4 h at 25°C in an automatic spectral scanning multimode reader (Varioskan, Ascent FL; Labsystems, Finland), and fluorescence emission measured at 590 nm as relative fluorescence units (RFUs) (excitation at 560 nm).

### Effect of Peptide Treatment on Bacterial and Fungal Infections in Tomato Plants

The efficacy of peptides in controlling infections by the bacterial and fungal plant pathogens was evaluated in potted tomato plants under greenhouse conditions. Tomato plants cv. Rio Grande were grown in 500 ml plastic pots in the greenhouse and were fertilized one time every week with 200 ppm of water-soluble NPK (20:10:20). Disease was determined in leaves of plants that have been sprayed with aqueous solutions of BP178, flg15, or BP100 at 125 μM. Streptomycin (0.10 mg/ml) was used as a reference control product, and water-sprayed plants were used as non-treated controls. Treatments were applied 24 h before pathogen inoculation. Pathogens were applied by spraying the corresponding suspensions until drop-off, and plants were incubated in the controlled environment greenhouse at 23 ± 2°C and a photoperiod of 16 h of light and 8 h dark and 60% relative humidity. The experimental design consisted of three biological replicates of three plants per each treatment and pathogen. The experiment was conducted two times.

After incubation, disease symptoms were allowed to develop, and the intensity of the infections was scored 10 days after pathogen inoculation, using a severity index ranging from 0 to a maximum of 4 (0, no symptoms; 1, necrosis/lesions up to 25% of the leaf surface; 2: necrosis/lesions on 25-50% of the leaf surface; 3, severe necrosis/lesions on 50–75% of the leaf surface; and 4, severe necrosis/lesions on >75% of the leaf surface). In every plant, each of the seven leaves (each with 4–5 leaflet) was rated according to the index, and it was used to calculate a disease severity index per plant according to the formula:


$$\begin{array}{l} S=\overset{n}{\underset{i=1}{\sum }}\frac{Ii}{\left(n.4\right)}.100 \end{array}$$

where S is the severity of the infections per plant, I_i_ is the severity index for each leaf, n is the number of leaves measured, which is multiplied by the maximum severity index (i.e., 4). Then, the mean of the three plants for each biological replicate was used for the statistical analysis. Data set were subjected to analysis of variance (one-way ANOVA) to determine if there were significant differences between treatments in bacterial- and fungal-disease control. Efficacy of each treatment was calculated based on the severity of the treatment in relation to severity observed in the plants NTC group according to the formula:


$$\begin{array}{l} E\;\left(\%\right)=\frac{S_{NTC}-S_{Treatment}}{S_{NTC}}x\;100 \end{array}$$

### Plant Materials, Treatments, and RNA Extraction for Gene Expression Analysis

Seeds of tomato plants cv. Rio Grande were sown in hydroponic seed plugs (rockwool), germinated and grown under controlled greenhouse conditions (25 ± 2°C, 16-h light/15 ± 2°C, 8-h dark, and 60% RH). Two-week-old seedlings (two cotyledons) were transplanted into Rockwool plugs (7.5 × 7.5 × 6.5 cm, Grodan Ibérica). The experimental design consisted of three biological replicates of 10 plants per replicate (30 plants per treatment) and treatments with BP178, BP100, flg15, and SA, JA, and ethylene that were included as positive controls of defense-signaling pathways.

After 2 weeks from transplanting, plants were sprayed with aqueous solutions of BP178, BP100 or flg15 at 125 μM, SA, and JA at 2.5 mM (Sigma-Aldrich, St. Louis, MO, USA) to the run-off point. For the ethylene treatment, plants were enclosed in a sealed chamber and exposed to ethylene obtained by reacting ethephon (1 mM) (Nufarm España, Spain) with a disodium hydrogen phosphate buffer (2.5 mM) (Zhang and Wen, 2010). The concentrations of the peptides BP100 and BP178 were chosen on the basis of the concentrations that were found effective against infections by plant pathogens observed *in planta* assays that were previously reported (Badosa et al., 2017; Caravaca-Fuentes et al., 2021). In the case of SA, JA, and ethylene, the concentrations were selected because they were used in other reports on topical application of defense elicitors in plants (Reignault and Walters, 2007; Rivas-San and Plasencia, 2011; Zhang et al., 2011).

Control plants were treated with distilled water. About 24 h after product application, leaf samples were collected, immediately frozen in liquid nitrogen, and stored at −80°C.

For total RNA extraction, the plant material was ground to a fine powder in liquid nitrogen with the Tissuelyzer II system (Qiagen, Hilden, Germany). Total RNA was extracted from leaves using TriZol® (Invitrogen, Life Technologies) according to the manual of the manufacturer. Following the extraction protocol, RNA samples were routinely subjected to DNAse treatment (Ambion® Turbo DNA-free™, Life Technologies, Thermo Fisher Scientific) to remove any contaminant DNA. In each step, RNA was quantified at 260 nm using a Nanodrop N-2000 spectrophotometer (Nanodrop Technologies LLC, Wilmington, DE, USA), and its integrity and quality verified by denaturing agarose gel electrophoresis and OD 260/280-nm absorption ratios, respectively. RNA samples of 10 plants were pooled in the same Eppendorf tube, and three biological replicates per treatment were analyzed (30 plants/treatment). This RNA was used as starting material to analyze the expression profiles of treated plants.

### Microarray Analyses

The GeneChip™ Tomato Gene 1.0 ST Array (Affymetrix, Thermo Fisher Scientific) was used for comparing transcriptomes from plants treated with BP178 and flg15. In addition, plants treated with the reference products SA, JA, and ethylene, as well as non-treated control plants were included in the analyses. The tomato GeneChip contains 37,815 probe sets to analyze 715,135 transcripts (20–25 probes per gene). Three GeneChips were used to analyze three biological replicates per treatment (three replicates x 10 plants). About 1 μg of DNAse-treated RNA was sent to the Unit of Genomics at the Complutense University of Madrid for cDNA synthesis, labeling, hybridization to whole transcriptome array, washing, scanning, and data collection. High-quality RNA was subjected to the GeneChip® WT Plus Reagent Kit (Affymetrix) that is used to prepare RNA samples for whole transcriptome expression analysis. Briefly, the integrity of the RNA samples was tested in the Agilent Bioanalyser (Agilent Technologies Inc., Sta. Clara, CA, USA) and used to synthesize double-stranded cDNA. After *in vitro* transcription (IVT) reaction in the presence of biotinylated UTP and CTP, a biotin-labeled cRNA was generated from the double-stranded cDNA. The cRNA is cleaned and fragmented into sequence of about 100 nucleotides, labeled using TdT, and hybridized to the Tomato Gene 1.0 ST Arrays. Subsequently, chips were washed and fluorescence stained with phycoerythrin using the antibody amplification step described in the GeneChip™Fluidics Station 450 (Thermo Fisher Scientific), and fluorescence was quantified. After sample scanning, data were extracted, background-adjusted and normalized intensities of all probes were summarized into gene expression by the GeneChip Expression Console Software (Affymetrix, Thermo Fisher Scientific), using the Robust Multichip Average (RMA) algorithm (Irizarry et al., 2003). Preprocessed data were analyzed by the web-based Babelomics (Medina et al., 2010) for gene expression analysis as the ratio of normalized fluorescence value between two compared treatments. This ratio was then scaled using base 2 logarithm to obtain the log2 ratio, which, in absolute terms, is known as fold-change. Sequences showing expression changes higher than 2-fold change (fold change, FC), and with FDR-adjusted p value below 0.05, were considered to be differentially expressed.

Overexpressed genes were functionally annotated using the gene function analysis tools included in the PANTHER classification system (v. 14.0) and/or in the SOL Genomics Network.

### Quantitative Real-Time PCR Analyses

To validate the expression patterns detected by microarray analyses, we analyzed a total of 14 *Solanum lycopersicum* genes encoding proteins involved in plant defense mechanisms (Table 1). These genes showed different fold change patterns, including upregulation and no significance changes after BP178 treatment. Oligonucleotide primers were designed according to the nucleotide sequence available at the Sol Genomics Network (ITAG release 2.40) using Primer Designing Tool included in the NCBI database. The reference gene actin was used as an internal control. Primers and the tomato genes implicated in plant defense response are listed in Supplementary Table 1.

**Table 1:** Associated functions to overexpressed defense related genes, according to RT-qPCR, in tomato plants in response to BP178 treatment.

**Gene**	**Inducing agent/pathway**	**Molecular function/property**	**References**
*PR3, Chi and Chi.2*	Abiotic agents (ethylene, salicylic acid, salt solutions, ozone, UV light) and by biotic factors (fungi, bacteria, viruses, viroids, fungal cell wall components, and oligosaccharides)	Carbohydrate metabolic process, acting on fungal cell wall degradation.	Sharma et al., 2011, Grove, 2012
*PR1, Pathogenesis-related protein-1*	Biotic agents/Salicylic acid	Marker for SA-acid mediated response and SAR in tomato	van Loon and van Strein, 1999, Chen et al., 2014
*Harp, Harpin-induced protein-like*	Plant defense responses, biotic agents	Multifunctional proteins	Zhang et al., 2011
*PR9, Peroxidase 1*	Biotic agents/Salicylic acid	Strengthening plant cell walls by catalyzing lignin deposition	Ebrahim et al., 2011 Taheri and Tarighi, 2012
*ERF, Ethylene responsive transcription factor*	Biotic and abiotic agents/Ethylene	Transcription factor activity, sequence-specific DNA binding	Müller and Munné-Bosch, 2015
*BCB, Blue-copper-binding protein gene*	Defense related responses	Protein binding. Oxidation/reduction process	Hao et al., 2015
*OLP, Osmotin-like protein, PR5*	Abiotic agents (salt, drought, cold) and biotic agents (fungi)	Protein binding, interaction with transcription factors involved in SA-dependent activation PR-genes. Stress-responsive multifunctional protein. Provides osmotolerance to plants.	Patade et al., 2013, Hao et al., 2015, Chowdhury et al., 2017
*PR7, P69G, Subtilisin-like protease*	Response to biotic and abiotic agents	Serine-type endopeptidase activity. Involved in signaling cascades.	Figueiredo et al., 2018

For each gene system, the concentration of the primer pair was optimized to prevent nonspecific reactions or artifacts that could hide the real result. Melting (dissociation) curve analysis was performed after each amplification to confirm the specificity of the amplified product/to prevent the detection of artifacts (as described in Badosa et al., 2017).

Gene expression analysis was performed by Quantitative Real-Time PCR (RT-qPCR). First-strand of complementary DNA (cDNA) was generated from leave RNA using reverse transcriptase (High Capacity cDNA Reverse Transcription Kit, Invitrogen) according to the manual of the manufacturer. This cDNA product was generated from each sample and was assayed for quantification of the expression levels of each of 25 tomato genes.

Quantitative Real Time-PCR was carried out in a fluorometric thermal cycler (7300 Real-Time PCR System, Applied Biosystems®, Waltham, MA, USA) using the Mix SYBR® Green PCR Master Mix (Applied Biosystems) as described in Badosa et al., 2017. The total reaction volume was 20 μl containing 1x Sybr Green Master Mix (Applied Biosystems), the optimized concentration of primers (final concentration of 300 mM for LePPO-f/LePPO-r, LeGLUA-f/LeGLUA-r, and LeAct-f/LeAct-r primer pair; 100 mM for the rest of primers used in this study) and 2 μL of RT reaction (cDNA). qPCR conditions were as follows: (1) an initial denaturation step (10 min at 95°C); (2) amplification and quantification (50 cycles of 15 s at 95°C and 1 min at 60°C); and a melting curve program (60-95°C with a heating rate of 0.5°C/s) as described in Badosa et al. (2017). Reactions were carried out in duplicate in 96-well plates. Controls from no cDNA template were included as negative controls. The relative quantification of each individual gene expression was performed using the 2^−ΔΔCt^ method (Livak and Schmittgen, 2001). Relative expression values of each plant defense were calculated normalizing against the tomato *actin* gene as an internal control. Statistical significance was determined using the REST2009 Software (Pfaffl et al., 2002).

## Results

### Antimicrobial Activity

Antibacterial and antifungal activity of BP178, flg15, and BP100 are shown in Table 2. BP178 and BP100 exhibited strong activity against Pto and Xcv. Specifically, BP178 showed a minimal inhibitory concentration (MIC) < 1 μM against Xcv and between 1 and 10 μM against Pto. The parent peptide BP100 showed MIC values, ranging from 1 to 10 μM against both bacterial pathogens. In contrast, the antifungal activity of BP178 and BP100 against Bc was very low, with MIC values ranging between 25 and 100 μM. Peptide flg15 was neither antibacterial nor antifungal at the maximum dose tested (100 μM).

**Table 2:** Sequence, number of amino acids, charge, and antimicrobial activity of the peptides used in this study.

				**Antimicrobial activity MIC^a^ (μM)**		
				**Bacteria**		**Fungi**
**Code**	**Sequence**	**#Aa^b^**	**Total net charge**	***Xcv***	***Pto***	***Bc***
BP178	KKLFKKILKYL–AGPA–GIGKFLHSAK–KDEL-OH	29	7	<1	1–10	50–75
BP100	KKLFKKILKYL-NH_2_	11	5	1–10	1–10	25–50
flg15	RINSAKDDAAGLQIA-OH	15	0	>100	>100	>100

a *Minimal inhibitory concentrations (MICs) were determined against Xanthomonas campestris pv. vesicatoria (Xcv), Pseudomonas syringae pv. tomato (Pto), and Botrytis cinerea (Bc)*.

b *Number of amino acids*.

**Figure 1:**
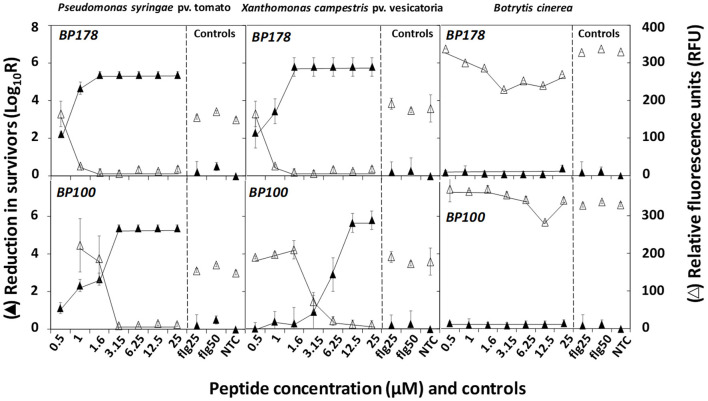
Effect of peptides BP178 and BP100 in cell survival (black triangles) and resazurin cell viability (white triangles) of *Pseudomonas syringae* pv. tomato, *Xanthomonas campestris* pv. vesicatoria, and *Botrytis cinerea* after exposure to the peptides for 60 min. Controls of flg15 at 25 (flg25) or 50 μM (flg50) and non-treated (NTC) were included. Values are the means of three replicates, and error bars represent standard deviation of the mean.

The bactericidal and fungicidal activities as determined by the contact and resazurin tests (cell survival and cell viability, respectively) are shown in Figure 1. BP178 led to a decrease in the survival of Xcv and Pto of 2.29 log reduction (N_0_/N) at 0.5 μM, which increased to 5.5 at 1.6 μM. For BP100, a maximum Pto and Xcv survival reduction of 5.4 and 5.7 log was observed after incubation at 3.2 and 12.5 μM, respectively. BP178 and BP100 practically showed a very slight fungicidal activity against Bc. As expected, flg15 did not reduce bacterial or fungal survival. The resazurin test confirmed the findings on cells survival, because survival was inversely related to resazurin cell viability (y = – 0.2401x + 2.4557, R^2^ 0.892) (Supplementary Figure 2).

### Effect of Peptides Treatment of Tomato Plants on Bacterial and Fungal Infections

**Figure 2:**
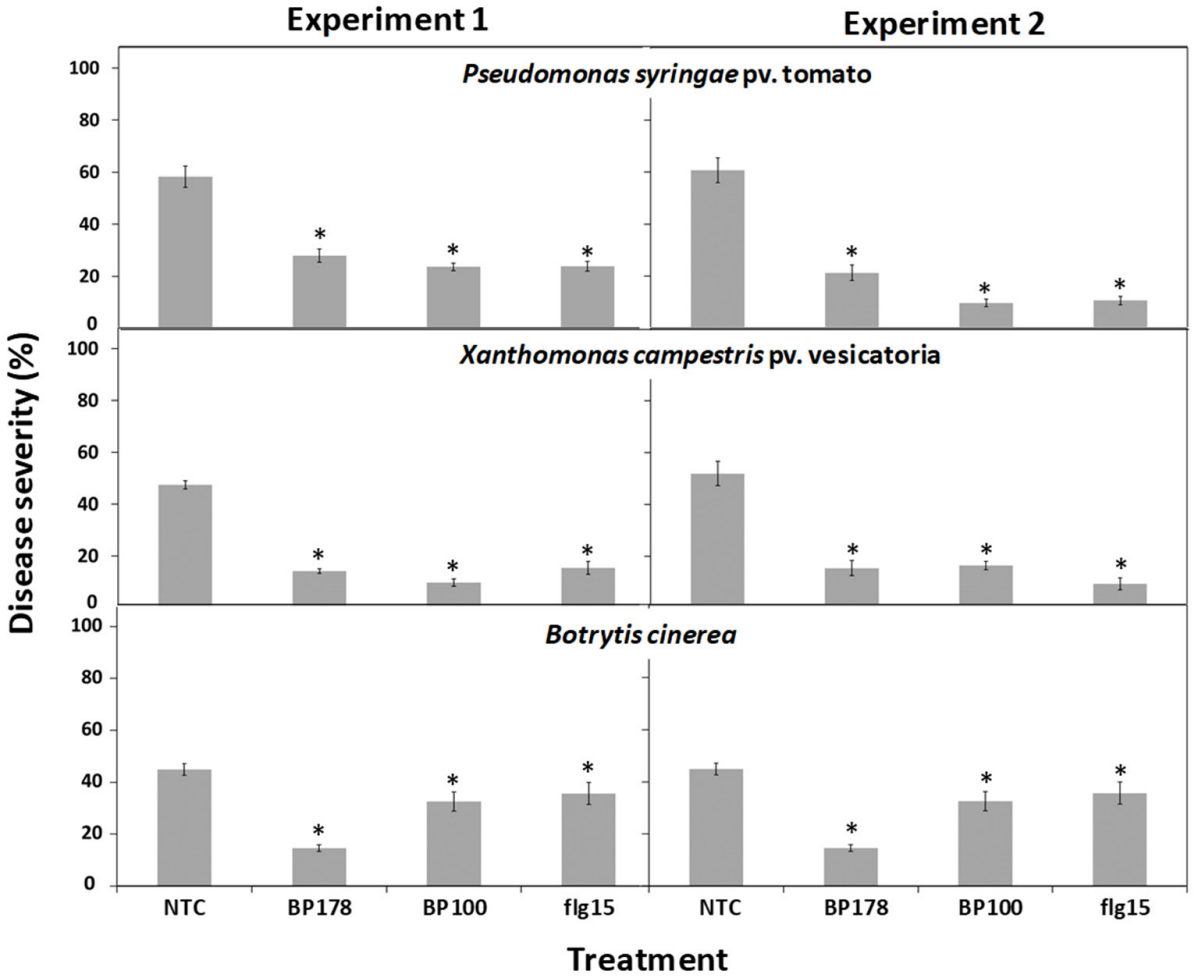
Protection of tomato plants against bacterial and fungal infection after topical treatment with BP178 in comparison with the parent peptide BP100 and flg15. Two independent assays were performed, and peptides were applied at 125 μM by spraying plants 24 h before pathogen inoculation. Disease severity was evaluated on tomato plants 10 days after pathogen inoculation (10^7^ ufc/ml for bacterial pathogens; 2.5 × 10 ^5^ conidia/ml for *B. cinerea*). Values correspond to the mean disease severity of three replicates of three plants per each treatment. Standard errors are indicated on bars. The asterisk denotes statistically significant differences with non-treated control plants (NTC) (Tukey's test, *p* > 0.05).

The results of the effect of treatments were consistent but slightly different between the two experiments performed. The preventive spray of peptide BP178 on tomato plants inhibited infections caused by Xcv, Pto, and Bc (Figure 2). More in detail, after treatment, disease severity in bacterial speck (Pto) was 21.3 and 27.9% for the two experiments performed (52.1 and 64.9% efficacy), and, in bacterial spot (Xcv), it was of 14.2 and 15.5 (around 70% efficacy), compared with non-treated controls (58.2% in experiment 1 and 60.8% in Pto in experiment 2, and 47.5% in experiment 1 and 51.9% in the second experiment in Xcv). The effect of BP100 and flg15 was similar to BP178 against Pto and Xcv infections.

In the case of Bc, disease severity due to the BP178 treatment was 14.6 and 29.4% for the two experiments (67.4 and 38% efficacy), compared to non-treated controls (47.5% in experiment 1 and 44.9% in experiment 2). Interestingly, there was a slight effect, but significant, of BP100 and flg15. This result greatly contrasts with the slight antifungal activity of BP178, BP100, and flg15 *in vitro*.

### Effect of Peptide Treatments on the Expression of Defense-Related Genes in Tomato

#### Microarray Analysis

The analysis revealed that of the 37,815 genes in the tomato microarray, the treatments modified the expression of several genes, following different patterns. According to the criteria for upregulation (fold change (FC ≥ 2) and downregulation (FC ≤ 0.5), the expression was modified in: 112 genes in BP178 (100 upregulated, 12 downregulated), 191 genes in flg15 (160 upregulated, 31 downregulated), 2,974 genes in SA (1,534 upregulated, 1,440 downregulated), 2,236 genes in JA (1,122 upregulated, 1,114 downregulated) and 1,280 in ethylene (826 upregulated, 454 downregulated). A detailed list of the differentially expressed genes for BP178, flg15, SA, JA, and ethylene treatments is given in Supplementary Table 2.

After the BP178 treatment, a total of 100 genes were upregulated (more than 2-fold) in comparison to the non-treated control. A set of 90 genes was functionally annotated, while the remaining 10 transcripts had unknown function or had no available hit. From the annotated genes, 74.4% of transcripts were identified as defense-related genes (67 out of 90 mapped ID), sharing homology with transcription factors (WRKY, MYB, and NAC), signal transduction genes (ethylene responsive transcription factor (ERF), serine/threonine protein-kinase), hormone-related genes, lipoxygenases, harpins, acetyltransferases, cytochrome P450, and several well-known pathogeneses-related genes (Table 3). PR-genes overexpressed after BP178 treatment, coded for antifungal/antimicrobial proteins (PR1), β-1,3-glucanases (PR2), chitinases (PR3, PR4), thaumatin-like proteins (PR5), endopeptidases inhibitor (PR6), subtilisin-like proteins (PR7), peroxidases (PR9), ribonuclease-like proteins (PR10), and lipid-transfer protein (PR14). The number of highly overexpressed genes (FC > 4) was 22, where the maximum FC values were reported in lipoxygenases (FC 14.01), endochitinases (FC 7.36), and lipid-transfer proteins (FC 7.18).

**Table 3:** Relevant upregulated (2-fold or higher; FDR< 0.05) transcripts after BP178 treatment (125 μM), identified in this study, associated with plant-defense response (GO term GO:0006952).

**Family/superfamily**	**Gene accession No**.	**BP178 *vs* NTC (FC)**	**Property/ GO molecular function**	**GO biological process**
Blue copper protein, Plastocyanin-like	Solyc03g116690 Solyc03g116700	2.41; 3.64	Copper ion binding, electron transfer activity	Redox reactions occurring during primary defense responses.
Homeobox-like domain	Solyc02g087960 Solyc04g005800	2.33; 2.17	DNA-binding transcription factor activity	Responses to biotic and abiotic stresses.
AP2/ERF transcription factor	Solyc09g089930 Solyc04g078640 Solyc12g056980 XM004244583	3.38; 2.46; 2.34; 2.82	Transcription regulatory region DNA binding	Defense response. Ethylene and JA signaling pathways.
NAC transcription factor	Solyc05g007770	2.82	Transcription regulatory region DNA binding	Response to stress, cold and drought stress and methyl methanesulfonate (MMS) treatment.
Mitochondrial peptide methionine sulfoxide reductase	Solyc02g063250	2.54	Oxidoreductase	Response to oxidative stress.
Lipoxygenase	Solyc08g029000	14.04	Lipoxygenase	Pest resistance and senescence. Responses to wounding. Involved in hypersensitive response.
Peptidase C1	Solyc02g077040	2.66	Cysteine-type endopeptidase	Hypersensitive response. Defense response to fungus, UV-B and to copper ion.
Cytochrome P450	Solyc09g066400 Solyc11g069800 Solyc04g078290	5.18; 2.09; 2.89	Oxidoreductase activity	Induction by ethylene. Involved in the biosynthesis of hormones and defensive compounds.
Ser/Thr protein kinase	Solyc10g045610 Solyc09g061410 Solyc12g005720	2.33; 2.15; 6.40	Receptor serine/threonine kinase binding	Signaling during pathogen recognition. Activation of plant defense responses.
Harpin-induced 1	Solyc02g036480	3.18	Role in plant immunity	Defense response to bacterium, virus, SA, wounding and hypoxia.
WRKY group III	Solyc08g082110	2.12	Transcription regulatory region DNA binding	Defense response to bacterium, chitin, water deprivation and SA. Regulation of JA mediated signaling pathway.
Acetyltransferase	Solyc02g064690 Solyc00g272810 Solyc08g068730	2.15; 2.56; 4.26	N-acetyltransferase activity	Response to ethylene and JA. Induced in response to pathogen infection, wounding, or elicitor treatments.
Bulb-type lectin domain	Solyc07g062490	5.38	Carbohydrate binding	Up-regulated by fungal elicitor, heat and cellular response to hypoxia.
Major facilitator superfamily (MFS) transporter	Solyc01g096720	3.70	Potassium ion antiporter activity	Response to water deprivation.
Peptidase A1	Solyc08g068870	3.00	Aspartic-type endopeptidase	Up-regulated locally and systematically during systemic acquired resistance (SAR) and locally by SA. Acts downstream of SA to suppress systemic immunity.
Isoprenoid synthase domain	Solyc03g006550	4.53	Terpene synthase activity/(E,E)-geranyllinalool synthase activity	Response to bacterium, herbivore, JA, wounding, singlet oxygen.
PR STH-2-like, BetVI	Solyc09g090980	5.56	Protein phosphatase inhibitor/signaling receptor activity	Response to biotic stimulus.
PR1	Solyc00g174330 Solyc01g106620	2.56; 2.84	Antimicrobial, fungicide	Defense response to fungus, response to biotic stimulus.
PR2	XM004228957	3.18	β-1,3-Glucanase	Defense of plants against pathogens.
PR3	Solyc02g082920 Solyc05g050130 Solyc02g061770 XR183217	3.13; 2.82; 7.36; 2.02	Endochitinase (acidic endochitinase, also lysozyme activity)	Response to bacterium and wounding, defense response to fungus, cold, water deprivation, wounding and to salt stress.
PR4	Solyc01g097270 Solyc04g072000	2.35, 5.32	Barwin domain chitinase I/II	Defense response to fungus and bacterium.
PR5	Solyc08g080660 Solyc08g080640	4.31; 4.08	Thaumatin like-proteins	Response to infection by a pathogen and possess antifungal activity. Induced by osmotic stress.
PR6	Solyc03g098740 Solyc08g080630 Solyc06g034370	2.43; 3.63; 3.87	Endopeptidase inhibitor	Response to wounding, herbivore, insects.
PR7, Peptidase S8 (subtilisin-like)	Solyc08g079870 XM004249457 Solyc08g079900	3.71; 2.63; 4.77	Serine-type endopeptidase	Pathogen recognition and immune priming.
PR9	Solyc07g056480 Solyc04g071890 Solyc09g011630	2.61; 2.50; 2.40	Peroxidase	Response to environmental stresses such as wounding, pathogen attack and oxidative stress.
PR10	Solyc05g007950	6.36	Ribonuclease like-proteins	Innate immune response. Essential role in Innate immune response by recognizing and degrading RNAs from microbial pathogens.
PR14	Solyc06g084190 Solyc08g007460 Solyc08g067550	2.23; 7.18; 3.88	Lipid-transfer protein	Components of the plant innate immune system. Responses to biotic and abiotic stresses and fungus.

*FC, fold-change value; SA, salicylic acid; JA, jasmonic acid*.

**Figure 3:**
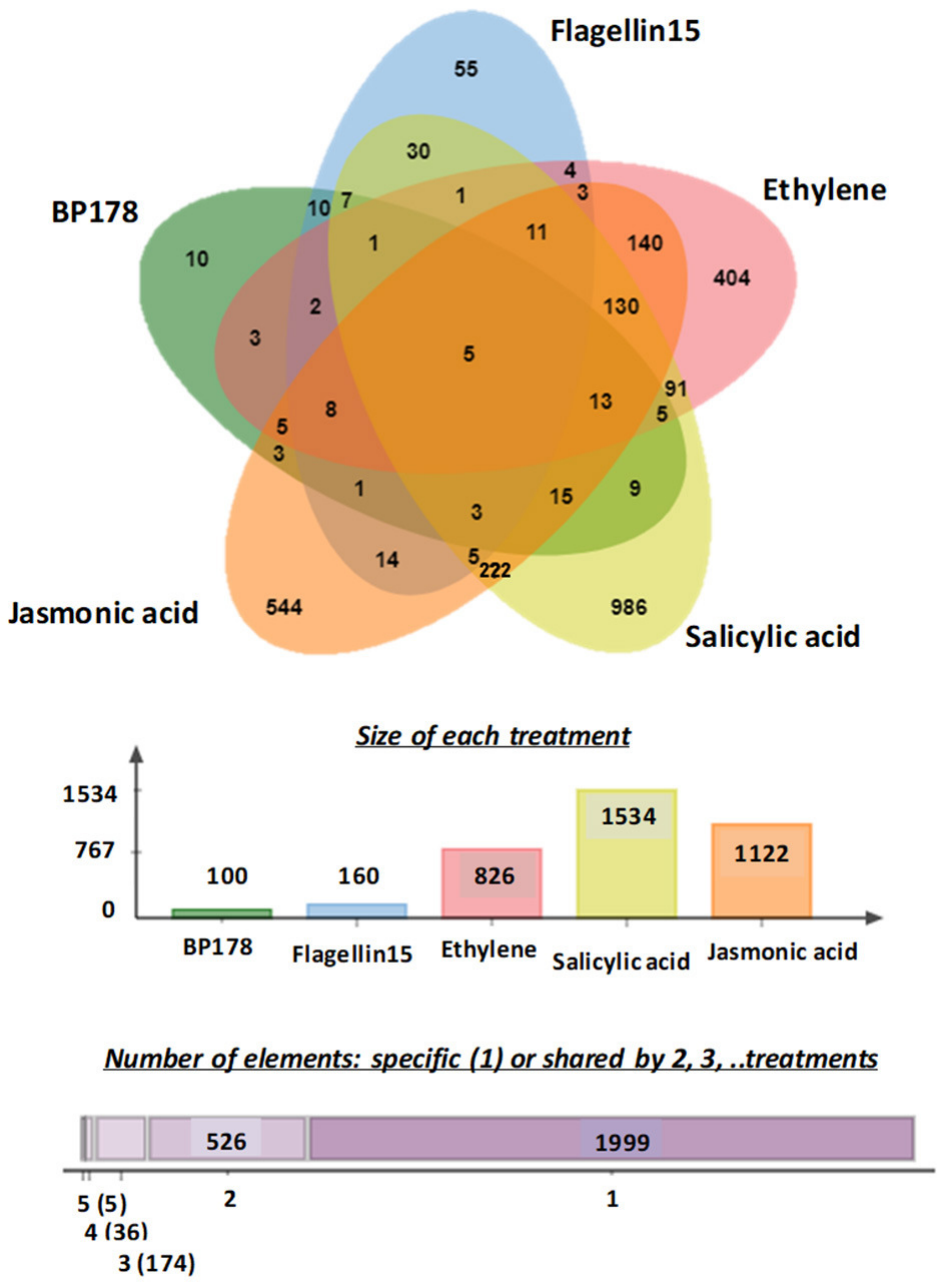
A Venn diagram of overexpressed genes in tomato plants after BP178, flagelin15, ethylene, salicylic acid, and jasmonic acid treatment. Overlapping regions of the circles indicate genes that are overexpressed in more than one treatment. Genes with fold-change above two were included in the analysis. The numbers in the graphic indicate the total numbers of overexpressed genes in each treatment. In the second chart, 1,999 overexpressed genes are specific of one list; 526 overexpressed genes are shared by two lists. Numbers in brackets represent the number of overexpressed genes shared by three, four, and five lists.

A Venn diagram (Bardou et al., 2014), to overlap differentially overexpressed genes after the treatments and to compare gene expression between response to BP178 and the other treatments, is shown in Figure 3. Among the BP178-upregulated genes, five genes were also induced after flg15, SA, JA, and ethylene treatment. Specifically, these transcripts corresponded to chitinase (PR4; FC 5.32), endochitinase (PR3; FC 3.16), a glycoprotein involved in signaling mechanisms (FC 5.38), acetyltransferase (FC 4.26), and hydrolase (FC 3.39). Except the hydrolase, all the other genes code for proteins directly involved in plant-defense responses. Ten genes were transcriptionally induced exclusively by the BP178 treatment, and seven of them can be mapped and identified as pathogenesis-related protein-1, glycosidase, a member of ABC transporter family, ser/thr protein kinase, cold shock protein (chaperone), pre-mRNA-splicing factor CLF1, and CXE carboxylesterase.

In addition, the Venn diagram revealed the commonly overexpressed transcripts in the five datasets (treatments). Within the 90 overexpressed and mapped genes after BP178 treatment, 37 were also overexpressed by flg15, 42 by ethylene, 58 by SA, and 53 by JA treatments (Figure 3).

The raw data of the microarray study are deposited in the National Center for Biotechnology Information (NCBI) repository, as metadata (experimental procedures for the transcriptomics analysis and experiment design) and the matrix data results for the different treatments. The code number at GEO webpage for the accession is GSE183707.

#### Quantitative Real-Time PCR Analyses

RT-qPCR was performed with 14 selected defense genes in order to validate the gene expression profile revealed by microarrays analysis in response to BP178 treatment. These candidate genes were chosen among genes showing significant induction profiles in the previous microarray analysis of *Solanum lycopersicum*, which encode proteins involved in plant-defense mechanisms (Supplementary Table 1) or with no significant changes in expression after the treatments.

**Figure 4:**
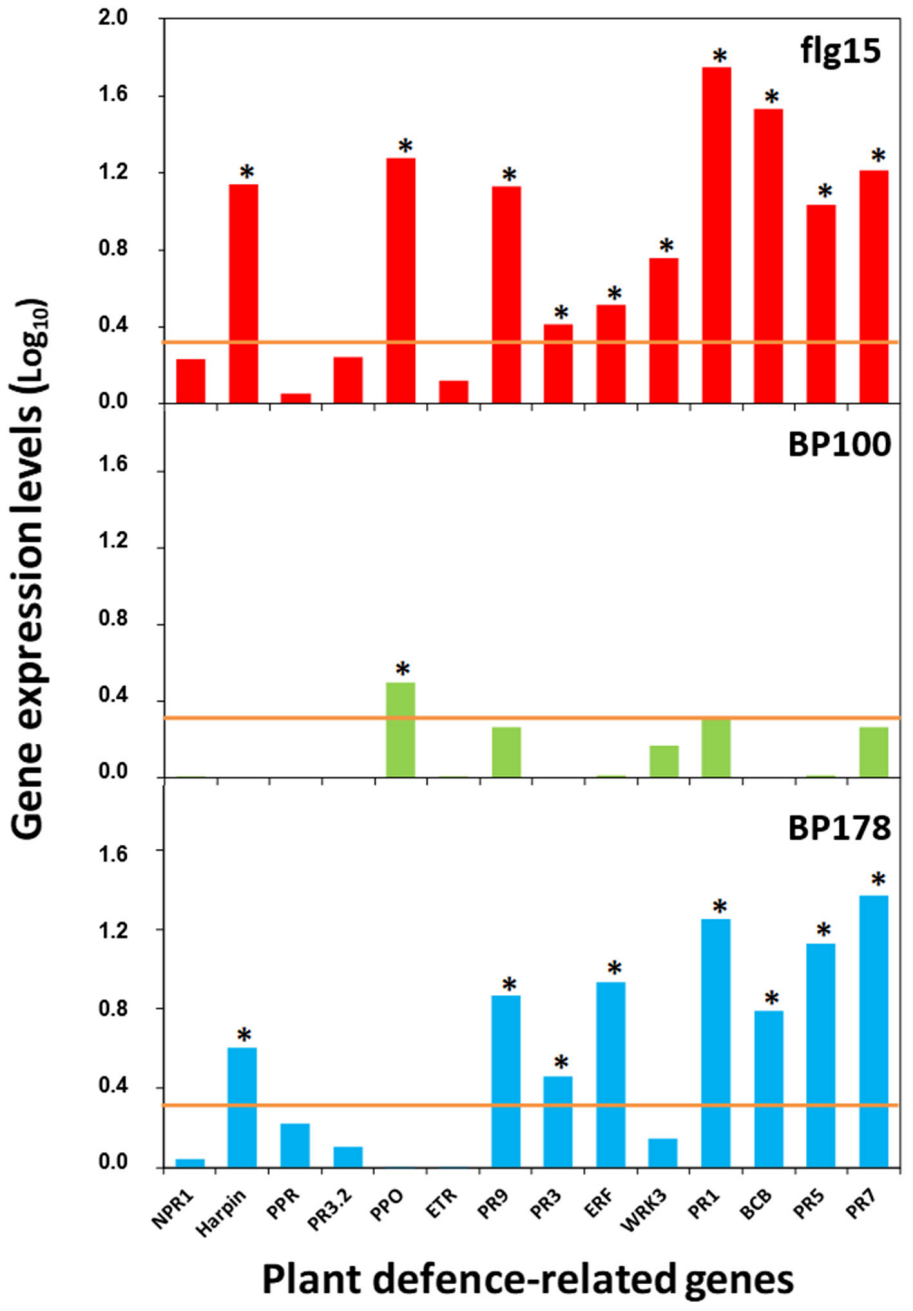
Relative expression levels (log_10_) of selected tomato plant-defense genes verified by qPCR analysis after treatment with the peptides. Orange line, cut-off values for gene induction are considered fold changes above 2 (log_10_, 0.3) (relative quantification using the ΔΔCt method). Asterisk, significant values of fold change. Gene expression data for BP100 and flg15 in the case of *PR1* gene have been previously published (Badosa et al., 2017).

A significant correlation was observed between the RT-qPCR and microarray data (Chi-square Pearson correlation coefficient of 0.789, *p* < 0.001, n = 70) (Supplementary Figure 3). Specifically, BP178 treatment induced overexpression of *harpin, PR9, PR3*, ERF, PR2, BCB, PR5, and PR7, similarly to the flg15 treatment that, apart from these genes, also overexpressed a polyphenol oxidase and the transcription factor WRKY3 (Figure 4). Contrarily, the treatment with the bactericidal peptide BP100 caused a slight overexpression of only one out of 14 genes (e.g., *polyphenol oxidase*).

## Discussion

Biostimulant application in agriculture represents a powerful strategy to improve both plant yield and tolerance to abiotic and biotic stresses (Rouphael and Colla, 2020). These products interact with plant-signaling cascades that triggered the expression of stress-responsive genes. Rapid responses to plant pathogens could trigger systemic signaling pathways and lead to plant resistance against pathogen attack (Moore et al., 2011; Wu et al., 2014). In the present study, we investigated the antimicrobial activity of peptide BP178 (Badosa et al., 2013; Montesinos et al., 2017) and its potential use as biostimulant to improve resistance to biotic and abiotic stresses in tomato, one of the major crops cultivated worldwide. In addition, the activity of BP178 was compared to the antibacterial peptide BP100 that does not have plant defense elicitation activity and to the plant-defense elicitor peptide flg15.

BP178 showed potent bactericidal activity against *Xanthomonas campestris* pv. vesicatoria and *Pseudomonas syringae* pv. tomato. In addition, we have shown here that BP178 applied by spraying to tomato plants was effective against infection by Pto, Xcv, and also Bc. These results agree with previous reports, indicating the effect against other plant pathogenic bacteria like *X. arboricola* pv. pruni, *Erwinia amylovora*, and *Xylella fastidiosa* (Badosa et al., 2013; Baró et al., 2020). However, the control of Bc infections in tomato was not expected due to the low *in vitro* antifungal activity exhibited by BP178. Therefore, we hypothesized a possible role of BP178 as a plant-defense elicitor. This possibility was previously pointed out because tobacco leaf infiltration with BP178 showed an HR-type response in tobacco plants, similarly to other hybrid peptides, incorporating BP100 (Badosa et al., 2013).

The treatment of tomato plants with BP178 and the subsequent analysis of microarray data revealed that 100 genes showed differential expression, compared to the non-treated control. Ninety of these genes were functionally annotated, and 74.4% were identified as defense-related genes. Furthermore, when the gene expression profile of tomato plants challenged with BP178 was compared to that of SA, JA, ethylene, and flg15 profile, several upregulated genes were found to be shared with these pathways. Flg15, as has been previously reported in pear plants (Badosa et al., 2017), triggered plant-defense responses, but has no antibacterial activity, whereas, contrarily, BP100 was strongly antibacterial, but had no significant gene induction activity according to the genes that were analyzed by RT-qPCR. Unfortunately, in the present work, the gene expression analysis of BP100 treatment was not included in the microarray, because we had previous evidence by RT-qPCR (Badosa et al., 2017; Oliveras et al., 2018) that, among 16 genes studied, only PinII and PPO were slightly overexpressed. Then, we cannot exclude that BP100 would induce the expression of genes other than the ones tested by RT-qPCR.

The present results are also in agreement with other reports involving flagellin (Zipfel et al., 2004; Pastor-Fernández et al., 2020). In addition, and as expected, we have found that tomato plants sprayed with SA, JA, or ethylene increased expression of a wide range of defense-related genes, but 10 genes were unique to BP178 challenged plants. Seven of these genes were mapped and identified as pathogenesis related protein-1, glycosidase, a member of the ABC transporter family, ser/thr protein kinase, cold shock protein, pre-mRNA-splicing factor CLF1, and CXE carboxylesterase.

Several pathways seem to be involved in BP178-triggered plant immunity, although pathways related to biotic stress were predominant. For instance, we found upregulation of genes coding for pathogenesis-related proteins like PR1, PR2, PR3, PR4, PR5, PR6, PR7, PR9, PR10, and PR14. This finding can be related to the decrease in severity of bacterial and fungal infections in tomato plants treated with BP178. The overexpression of PR genes was also reported as the reason to enhanced resistance in a variety of plants (i.e., potato, rice, grapevine, and tobacco) against a wide range of pathogens (Ali et al., 2018). Interestingly, it has been reported that the SA mediated activation, triggered after biotrophic/hemibiotrophic and necrothrophic pathogen attack, leads to expression of *PR1, PR2*, and *PR5* genes (Ali et al., 2018). In fact, the increased expression of *PR1* and *PR2* genes has been used as a molecular marker of the SAR pathway (Ceasar and Ignacimuthu, 2012), and the expression of *PR3, PR4*, and *PR12* genes is considered a signature of the JA pathway (Ali et al., 2018). Although both pathways follow different signaling systems, they can interact (Narváez et al., 2020), as we observed in BP178-challenged tomato plants.

The overexpression of the antifungal proteins PR2, PR3, PR4, and PR5 by BP178 treatment is particularly relevant since the plants are able to control infections caused by Bc, although this peptide has no significant *in vitro* antifungal activity. Interestingly, upregulation of PR3 and PR4 genes (chitinases) was reported in a *Fusarium*-resistant banana cultivar (Niu et al., 2018). Besides playing a key role against fungal pathogens, PR3 and PR4 also increase by other biotic factors, such as bacteria, viruses, viroids, or insects, and abiotic stresses, including osmotic, salt, cold, or wounding stresses, and salicylic acid and ethylene (Sharma et al., 2011; Grove, 2012). As mentioned above, the treatment with BP178 resulted also in the induction of *PR2, PR3*, and *PR5* genes involved in the ethylene-signaling pathway, in agreement with several studies reporting that ethylene perception and signaling are key factors in plant resistance to fungal and bacterial pathogens in many horticultural crops (Ravanbakhsh et al., 2018).

The pathogenesis-related gene *Osmotin*/*OLP* (coding a osmotin PR5 family) was highly induced in tomato plants in response to BP178 treatment. Osmotin overproduction has an effect against infection by several fungal plant pathogens, such as Bc (Monteiro et al., 2003), *Fusarium solani*, and *Colletotrichum gloeosporoides* (de Freitas et al., 2011), in agreement with our results of Bc infection control in tomato plants. In addition, it has been reported that the osmotin accumulated in plant cells in response to biotic or abiotic stresses (Chowdhury et al., 2017) provided osmotolerance, as well as induced cryoprotective functions (Barthakur et al., 2001; Goel et al., 2010). Moreover, the overexpression of the *osmotin* gene in transgenic plants results in enhanced tolerance to abiotic stresses, such as cold, salt, and drought (Patade et al., 2013).

Various *PR7* genes (subtilisin-like proteases, subtilases) were also overexpressed by the treatment of tomato plants with BP178. It is known that several PR7 proteins are specifically activated under different situations like after pathogen infection (Figueiredo et al., 2014) in tomato plants infected with citrus exocortis viroid (Granell et al., 1987), infection by *Pseudomonas syringae* or *Phytpohtora infestans*, and by SA treatment (Tornero et al., 1996; Jordá et al., 1999; Tian et al., 2005). In addition, subtilases are linked to immune priming in plants, and the DAMP systemin has been identified as one of the substrates of a subtilase (Schaller and Ryan, 1994, Kavroulakis et al., 2006). *PR7*s are also reported to be involved in abiotic stresses, such as drought and salt resistance mechanisms (Figueiredo et al., 2018).

Furthermore, plants challenged to BP178 overexpressed genes-coding PR10 proteins (ribonuclease-like proteins), which are known to confer activity against *Pseudomonas syringae* and *Agrobacterium tumefaciens*, among several pathogens (Ali et al., 2018). This finding is in agreement with the control of infections by Pto in tomato plants treated with BP178. Similarly, PR14 genes that were overexpressed in BP178 plants code for lipid-transfer proteins that exhibit both antibacterial and antifungal activities (Patkar and Chattoo, 2006).

In addition to the expression of several pathogenesis-related genes, BP178 induced several transcription factors, including ERF, WRKY, NAC and MYB, and enzymes implicated in cell wall and oxidative stress. ERFs are induced by SA, JA, and ethylene by integrating transcription factors and signaling pathways (Zheng et al., 2019). Our transcriptomic analysis with the microarray confirmed the overexpression of four *ERF* genes, and the RT-qPCR confirmed that BP178 almost triples the elicitor effect produced by flg15 on the *ERF* gene. ERFs are key regulators, integrating ethylene, abscisic acid, jasmonate, and the redox-signaling pathway in plant-defense response against abiotic stresses (Mizoi et al., 2012; Müller and Munné-Bosch, 2015). Moreover, BP178 challenged in tomato induced genes implicated in the synthesis of cytochrome P450, which is involved in plant steroid hormone biosynthesis (Farmer and Goossens, 2019).

Finally, the present study provides evidence that BP178 is a bifunctional peptide with bactericidal and defense-elicitor properties, protecting tomato from bacterial and fungal infections. This protection is partially due to the priming effect, similarly to flg15 that is conferred through very complex signaling pathways like the SA, JA, and ethylene. Interestingly, BP178 (C-terminal end) and flg15 (in the middle moiety) present a similar amino acid sequence [flg15: SAK-DDA (4-9 aa); BP178: SAKKDEL (23-29 aa)].

The singular properties of BP178, its biological performance, and the possibility to be produced using plants as biofactories (Montesinos et al., 2017), and, eventually, microorganisms, open great expectations for its future exploitation as a biopesticide for plant disease protection.

## Data Availability Statement

The datasets presented in this study can be found in online repositories. The names of the repository/repositories and accession number(s) can be found below: www.ncbi.nlm.nih.gov/, GSE183707.

## Author Contributions

EM, EB, MP, and LF obtained the financial support. LM, BG, LR, EB, and EM designed the research, analyzed the data, and wrote the paper. MP and LF provided the AMPs. LM, BG, and LR conducted and performed the experiments. All authors read, reviewed, and approved the final manuscript.

## Funding

This work was supported by the Spanish Ministerio de Economia y Competitividad (MINECO) Grant Nos. AGL2012-39880-C02-02 and AGL2015-69876-C2-1-R.

## Conflict of Interest

The authors declare that the research was conducted in the absence of any commercial or financial relationships that could be construed as a potential conflict of interest.

## Publisher's Note

All claims expressed in this article are solely those of the authors and do not necessarily represent those of their affiliated organizations, or those of the publisher, the editors and the reviewers. Any product that may be evaluated in this article, or claim that may be made by its manufacturer, is not guaranteed or endorsed by the publisher.
