# Supplementary material for: A Bifunctional Synthetic Peptide With Antimicrobial and Plant Elicitation Properties That Protect Tomato Plants From Bacterial and Fungal Infections
Source: Front Plant Sci. 2021 Oct 18;12:756357. doi: 10.3389/fpls.2021.756357 (PMC8558481; doi:10.3389/fpls.2021.756357)
Supplement: Supplementary file 1 [file Table_1.docx]

**Supplementary Table 1.** Genes and primers used for RT-qPCR analysis to quantify their expression levels in tomato plants in response to treatment with synthetic peptide BP178.

| **Gene** | **Primer code** | **Sequence (5’–3’)** | **Reference** |
| --- | --- | --- | --- |
| *PR3,Chitinase endochitinase family 19* | LeChi-f | TTCACCTTTGCTGACTGCCT | This study |
| (Solyc10g055820.1.1) | LeChi-r | CTTTGACAATTGCCGGGAGC |  |
|  |  |  |  |
| *Chi2, chitinase 2 family 18* | ChiAS-f | TTCGGCACTGATGGAAGTGG | This study |
| (Solyc11g072830.1.1) (PR3.2) | ChiAS-r | TTTTAAGCTTGCTACACGCGG |  |
|  |  |  |  |
| *PR7, P69G, Subtilisin-like protease* | LeSub1-f | ACCTAAAGGCGTTGTCGTGA | This study |
| (Solyc08g079900.1.1) | LeSub1-r | ACCCCAGACATTGAGCTGTT |  |
|  |  |  |  |
| *WRKY transcription factors* | LeWRK3-f | TTAGCAGCCGCCATATCAGG | This study |
| (Solyc03g116890.2.1) | LeWRK3-r | TGGCTGCCTGTCTTTCTCTG |  |
|  |  |  |  |
| *PR1, Pathogenesis-related protein-1* | LePR1A-f | TCTTGTGAGGCCCAAAATTC | Aimé et al., 2008 |
| (Solyc09g007010.1.1) | LePR1A-r | ATAGTCTGGCCTCTCGGACA |  |
|  |  |  |  |
| *PPO, Polyphenol oxidase* | LePPO-f | AGACGTAATTCCCACGTCCG | This study |
| (Solyc08g074650.2.1) | LePPO-r | GGCACGGTACACCGAAGTTA |  |
|  |  |  |  |
| *PR9, Peroxidase 1* | LePer1-f | TCTTAGCTGTTGCAGCTCGT | This study |
| (Solyc07g017880.2.1) | LePer1-r | CTAGTGTATGGCCACCGGAC |  |
|  |  |  |  |
| *NPR1 protein* | LeNPR1-f | AAAAGATCAAGCGGAGGCGA | This study |
| (Solyc02g069310.2.1) | LeNPR1-r | CTTTGACACTTGCACCGTCG |  |
|  |  |  |  |
| *ERF, Ethylene responsive transcription factor* | LeERT3-f | TCCGAAACAGTCACATCGCA | This study |
| (Solyc09g089930.1.1) | LeERT3-r | AGCATCTTCCGCGCTATCAA |  |
|  |  |  |  |
| *ETR6, Ethylene receptor* | LeETR-f | CCCGTTGTGTCCTCGTTACA | This study |
| (Solyc09g089610.2.1) | LeETR-r | CCGATGATCAAAGGCCAGGT |  |
|  |  |  |  |
| *OLP, Osmotin-like protein, PR5* | LeOsm2-f | TCCAATTCAATGCACAGCCA | This study |
| (Solyc08g080660.1.1) | LeOsm2-r | TAGGACCACATGGACCGTGA |  |
|  |  |  |  |
| *Harp, Harpin-induced protein-like* | LeHarp-f | ATTATGGCCCGTCCATTCCG | This study |
| (Solyc10g081980.1.1) | LeHarp-r | ATGCAATGACTCCGAGGACG |  |
|  |  |  |  |
| *PPR, Pentatricopeptide repeat protein* | Le5AS-f | GGTGCTTGTAGGGTCTACGG | This study |
| (Solyc09g090440.1.1) | Le5AS-r | CCATTCACCTCCACGATGCT |  |
|  |  |  |  |
| *BCB, Blue-copper-binding protein gene* | LeBCP-f | TTGGCACACACTGTCAAGGT | This study |
| (Solyc03g116700.2.1) | LeBCP-r | ACTGGCCAATAGGGTCGTTG |  |
| *Actin* | LeAct-f | CACTGTATGCCAGTGGTCGT | Badosa et al., 2017 |
| (Solyc04g011500.2.1) | LeAct-r | GACGGAGAATGGCATGTGGA |  |

**REFERENCES**

Aimé, S., Cordier, C., Alabouvette, C., and Olivain, C. (2008). Comparative

analysis of PR gene expression in tomato inoculated with virulent Fusarium

oxysporum f. sp. lycopersici and the biocontrol strain F. oxysporum

Fo47. *Physiol. Mol. Plant Pathol.* 73, 9–15. doi: 10.1016/j.pmpp.2008.

10.001

Badosa, E.,Montesinos, L., Cam,ó, C., Ruz, L., Cabrefiga, J., Francés, J., et al. (2017).

Control of fire blight infections with synthetic peptides that elicit plant defense

responses. *J. Plant Pathol.* 99, 65–73.
