# Supplementary figures and images for: A Bifunctional Synthetic Peptide With Antimicrobial and Plant Elicitation Properties That Protect Tomato Plants From Bacterial and Fungal Infections
Source: Front Plant Sci. 2021 Oct 18;12:756357. doi: 10.3389/fpls.2021.756357 (PMC8558481; doi:10.3389/fpls.2021.756357)

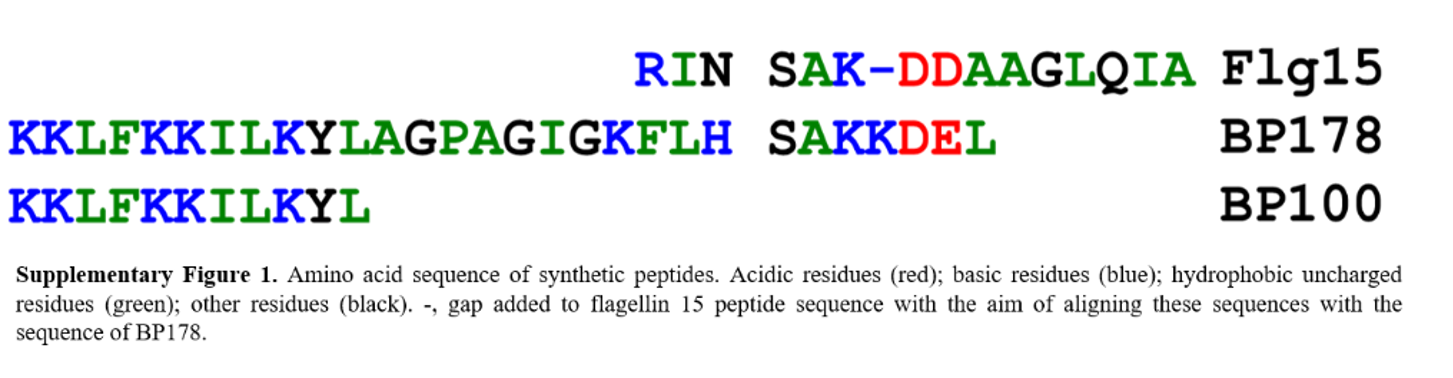

Supplement: Supplementary file 3 [file Image_1.TIF]

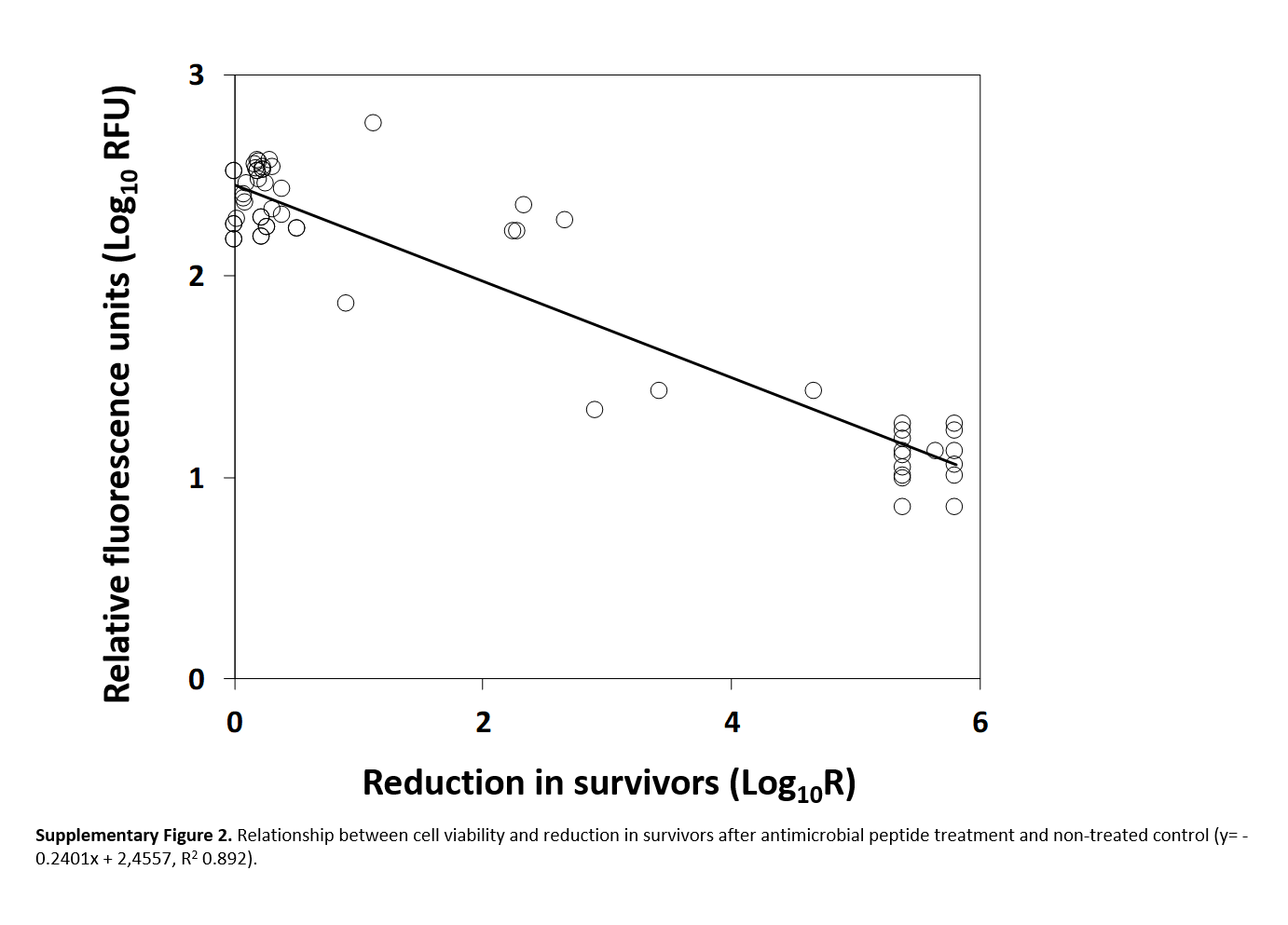

Supplement: Supplementary file 4 [file Image_2.TIF]

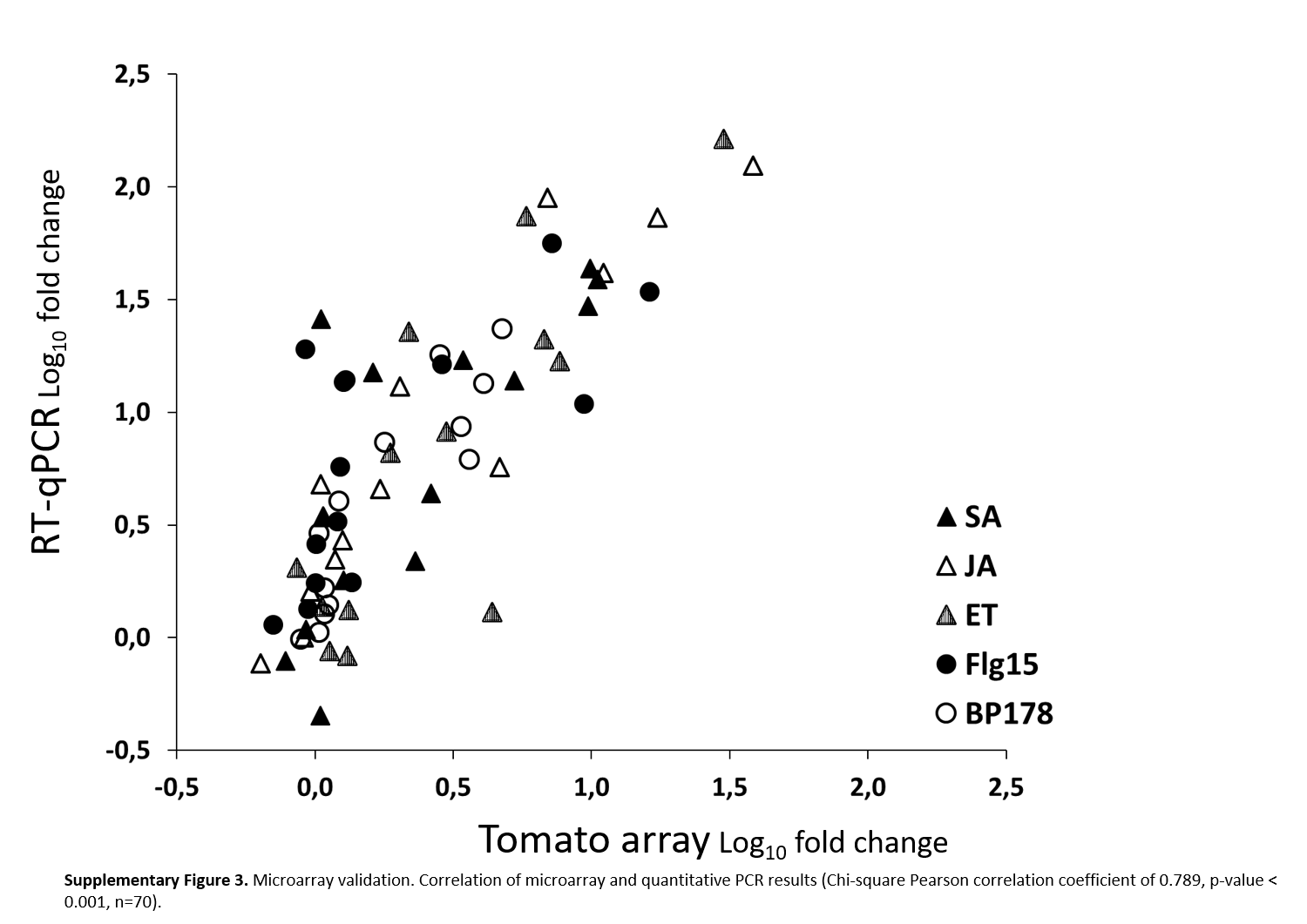

Supplement: Supplementary file 5 [file Image_3.TIF]
